# NT-proBNP trajectory after transcatheter aortic valve replacement and its association with 5-year clinical outcomes

**DOI:** 10.3389/fcvm.2023.1098764

**Published:** 2023-02-17

**Authors:** Yaoyao Zhou, Qifeng Zhu, Po Hu, Huajun Li, Xinping Lin, Xianbao Liu, Zhaoxia Pu, Jian’an Wang

**Affiliations:** ^1^Department of Cardiology, The Second Affiliated Hospital, Zhejiang University School of Medicine, Hangzhou, China; ^2^Department of Cardiology, Affiliated Jinhua Hospital, Zhejiang University School of Medicine, Jinhua, China

**Keywords:** aortic stenosis, transcatheter aortic valve replacement, NT-proBNP, trajectory, prognosis

## Abstract

**Background:**

There are only limited reports on the trends of NT-proBNP after transcatheter aortic valve replacement (TAVR) in aortic stenosis (AS) and even fewer report on the prognostic value of the NT-proBNP trajectory following TAVR.

**Objectives:**

This study aims to investigate short-term NT-proBNP trajectory following TAVR and explore its association with clinical outcomes in TAVR recipients.

**Methods:**

Aortic stenosis patients undergoing TAVR were included if they had NT-proBNP levels recorded at baseline, prior to discharge, and within 30 days after TAVR. We used latent class trajectory models to identify NT-proBNP trajectories based on their trends over time.

**Results:**

Three distinct NT-proBNP trajectories were identified from 798 TAVR recipients, which were named class 1 (*N* = 661), class 2 (*N* = 102), and class 3 (*N* = 35). Compared to those with trajectory class 1, patients with trajectory class 2 had a more than 2.3-fold risk of 5-year all-cause death and 3.4-fold risk of cardiac death, while patients with trajectory class 3 had a more than 6.6-fold risk of all-cause death and 8.8-fold risk of cardiac death. By contrast, the groups had no differences in 5-year hospitalization rates. In multivariable analyses, the risk of 5-year all-cause mortality was significantly higher in patients with trajectory class 2 (HR 1.90, 95% CI 1.03–3.52, *P* = 0.04) and class 3 (HR 5.70, 95% CI 2.45–13.23, *P* < 0.01).

**Conclusion:**

Our findings implied different short-term evolution of NT-proBNP levels in TAVR recipients and its prognostic value for AS patients following TAVR. NT-proBNP trajectory may have further prognostic value, in addition to its baseline level. This may aid clinicians with regards to patient selection and risk prediction in TAVR recipients.

## Highlights

### What is known?

The prognostic value of baseline natriuretic peptide levels has been validated in TAVR recipients.

### What is new?

This investigation investigated the prognostic value of NT-proBNP and its trajectories based on their trend over time using a large cohort of severe AS patients. Three distinct NT-proBNP trajectories were identified, and their association with 5-year clinical outcomes following TAVR was evaluated. Apart from the baseline level of NT-proBNP, its trajectory following TAVR might have clinical implications. It is valuable for risk prediction and concomitant therapy in AS population undergoing TAVR. Our findings implied different evolution of NT-proBNP levels in TAVR recipients and the necessity of reevaluating NT-proBNP following TAVR.

### What is next?

Further investigation is warranted to understand the underlying mechanisms and determine whether steps taken to mitigate the pathobiology resulting in elevated NT-proBNP levels may improve the clinical outcomes of TAVR recipients.

## Introduction

With recent advances in procedural techniques, patients with aortic stenosis (AS) benefit greatly from transcatheter aortic valve replacement (TAVR), a safe and effective treatment option compared to surgical aortic valve replacement (SAVR) (1). While unloading the heart by TAVR is critical for severe AS, less attention has been directed at what adjunctive therapies and clinical care might improve clinical outcomes alongside relieving the mechanical obstruction (2). Regardless of immediate outcome, 30% of TAVR recipients gain minimal symptomatic benefit, or die, within the first year after intervention (3).

Natriuretic peptides are cardiac neurohormones secreted by the myocardium in response to increased mechanical wall stress. Brain natriuretic peptide (BNP) and its pro-hormone N-terminal pro-BNP (NT pro-BNP) are reliable markers of AS and LV hypertrophy severity (4, 5). High circulating levels correlate with severe dyspnea symptoms [New York Heart Association (NYHA) class] and poor clinical outcomes in the AS population. Furthermore, baseline natriuretic peptide levels have been shown to predict survival and rehospitalizations after valve replacement. International practice guidelines advocate their role in risk assessment, prognostication, and therapy monitoring, particularly for patients with severe AS and equivocal symptoms (6).

However, several clinical trials and observational studies have shown controversial results and discordant findings regarding the association between baseline natriuretic peptide levels and mortality after TAVR (7). Prior studies, including a sub-analysis from the PARTNER 1 trial, failed to find an association between baseline BNP levels and post-TAVR prognosis (8). Post-TAVR change in BNP level, however, has been reported to predict clinical outcomes. Notably, most studies suggested that the association between baseline BNP and outcomes was a non-linear relationship, with interpretation hampered by inconsistency in cutoffs and the heterogeneity of TAVR recipients.

There is a paucity of data examining the relationship between NT-proBNP levels and post-TAVR prognosis. Nevertheless, most existing studies assessed natriuretic peptides only once (mostly at baseline), which neglects individual diversity in the impact of the TAVR and the course of the disease post-procedure (9, 10). There is a significant gap in the literature concerning the role and necessity of longitudinal assessments of NT-proBNP following TAVR (11). To enhance post-TAVR risk stratification and prognostication, assessing NT-proBNP over time might reveal more information not captured by a single evaluation.

We undertook an exploratory *post hoc* analysis to investigate whether NT-proBNP levels and their short-term trajectories are associated with clinical outcomes in patients with severe AS undergoing TAVR. In the present investigation, we hypothesized that NT-proBNP trajectories would constitute distinct phenotypic profiles that further enhance risk stratification in TAVR recipients.

## Materials and methods

### Study design and population

Patients with severe AS treated with TAVR at the Second Affiliated Hospital of Zhejiang University were prospectively enrolled into the Transcatheter Aortic Valve Replacement Single-Center Registry in the Chinese Population (TORCH) (NCT02803294). Patients were included from March 2013 to April 2021 if they had NT-proBNP levels recorded before TAVR (baseline), prior to discharge, and within 30 days after TAVR (Supplementary Figure 1). Patients with pure aortic regurgitation, conversion to open heart surgery or incomplete clinical data were excluded. The design, inclusion and exclusion criteria, definitions for clinical variables, and preliminary results of these trial and registry cohorts have been previously reported (12). The protocols were approved by the Medical Ethics Committee of the Second Affiliated Hospital of Zhejiang University, and all patients provided written informed consents.

### Clinical data and endpoints

Clinical data included baseline characteristics, procedural data, and follow-up outcomes obtained at baseline, hospital discharge, 30 days, 1 year, and 2 years. All data were collected from the local hospital database, scheduled outpatient clinic visits, or direct telephone interviews. NT-proBNP was measured from a blood sample and processed in standard fashion using a chemoluminescent immunoassay (Elecsys proBNP II; Roche, Minneapolis, Minnesota, United States).

Our analysis focused on the clinical endpoints of all-cause mortality, cardiovascular mortality, rehospitalization, and cardiovascular rehospitalization. Other clinical outcomes including: Mortality, rehospitalization, myocardial infarction, stroke, bleeding, new permanent pacemaker, new atrial fibrillation and renal dysfunction were defined according to the Valve Academic Research Consortium-3 criteria (13).

### Statistical analysis

We used latent class trajectory models to identify NT-proBNP trajectories over time. This is a specialized form of finite mixture modeling designed to identify latent classes of individuals following similar progressions of a determinant over time (14). Our models used second-order polynomials. After data standardization, we calculated the posterior probabilities of participants for each trajectory and then assigned participants *post hoc* to the trajectory with the highest probability. We estimated the best-fitting number of trajectories based on a minimum Bayesian Information Criterion while maintaining the posterior probabilities by class (>0.70) and class size (≥2% of the population) (15).

Continuous variables are presented as means and standard deviations (SD) and compared by Student’s *t*-test. Categorical variables are shown as percentages and frequencies and compared using the χ2 test or Fisher’s exact test, as appropriate. A two-sided *P* < 0.05 was considered statistically significant. We used multivariable logistic regression models to identify predictors of distinct NT-proBNP trajectories. The candidate variables were selected *a priori* for inclusion in the univariable logistic regression models Variates with *P* < 0.05 were then entered into a multivariate model to identify independent factors by the regression stepwise method. Time-to-first event curves are displayed using Kaplan–Meier estimates and compared by the log-rank test. Hazard ratios (HRs) and 95% confidence intervals (CIs) are estimated by Cox proportional hazards regression models. Adjustments were made for baseline variables [age, sex, body mass index (BMI), Society of Thoracic Surgeons (STS) score, diabetes, hypertension, chronic obstructive pulmonary disease (COPD), estimated glomerular filtration rate (eGFR), prior stroke, atrial fibrillation/flutter (AF), left ventricular ejection fraction (LVEF), NYHA class and NT-proBNP levels] and procedural complications (new or aggravated atrioventricular block, vascular complications, annular rupture, coronary obstruction, circulation collapse, aortic regurgitation paravalvular ≥ moderate and aortic regurgitation transvalvular ≥ moderate), and 30-day post-TAVR outcomes (NYHA ≥ Class III, myocardial infarction, stroke, disabling stroke, bleeding, life-threatening bleeding, new permanent pacemaker, new atrial fibrillation, and renal dysfunction). Covariates for this analysis were selected *a priori* based on historical prognostic relevance or clinical judgment, which were further selected in the multivariate analyses based on their statistical significance. Statistical analyses were performed using R statistical software (version 4.0.3).

## Results

### NT-proBNP trajectories

**FIGURE 1:**
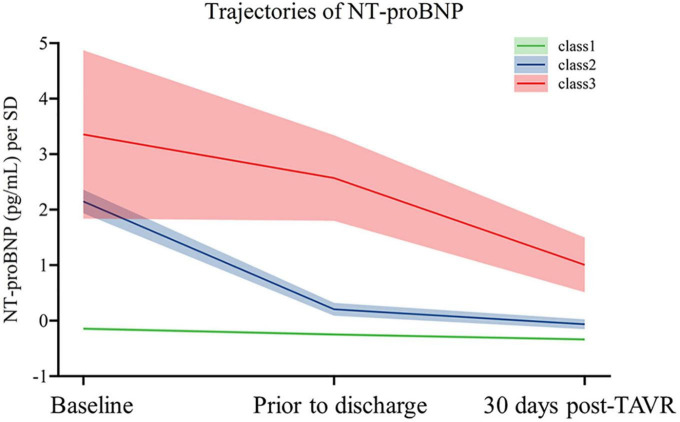
Trajectories of NT-proBNP of the study population. The figure shows trajectories of NT-proBNP levels from baseline to 5-year post-TAVR from 857 individuals. Shading around the lines represents confidence bands for the calculated trajectory.

A total of 798 patients were included in the final cohort. The median age was 75 [interquartile ranges (IQR) 70–80], and 42.9% were female. As Figure 1 depicts, we identified three distinct NT-proBNP trajectories based on their trend over time. Mean probabilities per trajectory ranged from 0⋅91 (SD 0.14) to 0⋅99 (SD 0.06). To facilitate interpretability, they were named class 1 (*N* = 661), class 2 (*N* = 102), and class 3 (*N* = 35), respectively. In patients with NT-proBNP trajectory class 1, baseline NT-proBNP was relatively low [1423.00 (466.00–3520.00)] and remained nearly unchanged throughout the first years. In contrast, baseline NT-proBNP levels were significantly higher in patients with NT-proBNP trajectory class 2 [17727.50 (13637.25–25312.75)] and class 3 [35000.00 (10078.00–35000.00)]. At the same time, the downward trends were remarkable in trajectory class 2 but not pronounced in trajectory class 3.

### Patient characteristics by NT-proBNP trajectories

Table 1 illustrates that patients with NT-proBNP trajectory class 1 were younger, had higher BMI, and were more likely to have atrial fibrillation and BAV at baseline. They also had better STS scores, NYHA Class, LVEF, and eGFR. On the other hand, patients with NT-proBNP trajectory class 3 were more likely to have higher STS scores, a history of PCI or MI, and lower baseline eGFR. Procedural complications and 30-day clinical outcomes after TAVR are shown in Table 2. Patients with NT-proBNP trajectory class 1 had the lowest risk of moderate to severe paravalvular regurgitation. In contrast, patients with NT-proBNP trajectory class 3 were more likely to suffer from perioperative circulation collapse and NYHA Class III or IV within 30-day post-TAVR. There were no between-group differences in other clinical characteristics.

**TABLE 1:** Baseline characteristics stratified by different trajectories.

Characteristic	Total	Class 1	Class 2	Class 3	*P*-value
	***N* = 798**	***N* = 661**	***N* = 102**	***N* = 35**	
Age (y)	75.00 (70.00–80.00)	74.00 (70.00–80.00)	76.00 (74.00–81.00)	76.00 (70.00–81.00)	0.017
Male (%)	456 (57.14)	374 (56.58)	61 (59.80)	21 (60.00)	0.78
BMI (kg/m2)	22.80 (20.30–25.00)	23.10 (20.80–25.30)	20.75 (19.02–23.40)	21.40 (19.65–24.35)	<0.01
Smoker (%)	142 (17.79)	122 (18.46)	14 (13.73)	6 (17.14)	0.51
STS score (%)	4.63 (2.79–8.30)	4.17 (2.55–7.08)	8.47 (5.28–12.35)	9.46 (5.79–15.03)	<0.01
Hypertension (%)	427 (53.51)	357 (54.01)	53 (51.96)	17 (48.57)	0.78
Diabetes (%)	164 (20.55)	142 (21.48)	18 (17.65)	4 (11.43)	0.26
AF (%)	131 (16.42)	98 (14.83)	27 (26.47)	6 (17.14)	0.01
COPD (%)	176 (22.06)	147 (22.24)	24 (23.53)	5 (14.29)	0.5
PVD (%)	109 (13.66)	86 (13.01)	15 (14.71)	8 (22.86)	0.22
Previous stroke (%)	36 (4.51)	28 (4.24)	6 (5.88)	2 (5.71)	0.62
Previous PCI (%)	95 (11.90)	75 (11.35)	9 (8.82)	11 (31.43)	<0.01
Previous MI (%)	11 (1.38)	8 (1.21)	0 (0.00)	3 (8.57)	0.01
Previous CABG (%)	2 (0.25)	2 (0.30)	0 (0.00)	0 (0.00)	0.99
NYHA Class					<0.01
1	11 (1.38)	11 (1.67)	0 (0.00)	0 (0.00)	
2	131 (16.42)	122 (18.46)	3 (2.94)	6 (17.14)	
3	400 (50.13)	360 (54.46)	28 (27.45)	12 (34.29)	
4	256 (32.08)	168 (25.42)	71 (69.61)	17 (48.57)	
NT-proBNP (pg/mL)	2117.50 (612.25–6182.75)	1423.00 (466.00–3520.00)	17727.50 (13637.25–25312.75)	35000.00 (10078.00–35000.00)	<0.01
eGFR (ml/min)	56.02 (42.09–73.22)	60.74 (46.09–76.73)	41.61 (30.65–51.42)	28.63 (12.39–40.61)	<0.01
LVEF (%)	58.90 (46.60–64.47)	60.50 (52.70–65.30)	39.60 (30.70–52.05)	42.00 (27.40–54.70)	<0.01
V max (m/s)	4.70 (4.21–5.26)	4.73 (4.24–5.30)	4.62 (4.09–5.18)	4.40 (3.90–4.86)	0.01
MPG (mm Hg)	51.00 (41.00–65.00)	52.00 (42.00–66.00)	47.50 (38.00–61.00)	41.00 (31.50–55.50)	<0.01
AVA (m2)	0.60 (0.46–0.77)	0.62 (0.47–0.78)	0.51 (0.40–0.68)	0.58 (0.42–0.78)	<0.01
BAV (%)	390 (48.87)	338 (51.13)	43 (42.16)	9 (25.71)	<0.01

Values are median (interquartile range), *n* (%), or mean SD.

AF, atrial fibrillation; AVA, aortic valve area; BAV, bicuspid aortic valve; BMI, body mass index; CABG, coronary artery bypass grafting; COPD, chronic obstructive pulmonary disease; eGFR, estimated glomerular filtration rate; LVEF, left ventricular ejection fraction; MI, myocardial infarction; MPG, mean pressure gradient; N, number; NYHA, New York Heart Association; PCI, percutaneous coronary intervention; PVD, peripheral vascular diseases; STS, Society of Thoracic Surgeons; V max, peak aortic velocity.

**TABLE 2:** Procedural complications and 30-day clinical outcomes after TAVR.

	Total	Class 1	Class 2	Class 3	*P*-value
	***N* = 798**	***N* = 661**	***N* = 102**	***N* = 35**	
**Procedural complications**					
New or aggravated AV block (%)	147 (18.42)	126 (19.06)	12 (11.76)	9 (25.71)	0.11
Vascular complications (%)	49 (6.14)	40 (6.05)	8 (7.84)	1 (2.86)	0.57
Annular rupture (%)	1 (0.13)	1 (0.15)	0 (0.00)	0 (0.00)	0.99
Coronary obstruction (%)	7 (0.88)	6 (0.91)	1 (0.98)	0 (0.00)	0.99
Circulation collapse (%)	27 (3.38)	16 (2.42)	6 (5.88)	5 (14.29)	<0.01
AR paravalvular ≥ moderate (%)	50 (6.27)	36 (5.45)	11 (10.78)	3 (8.57)	<0.01
AR transvalvular ≥ moderate (%)	5 (0.63)	3 (0.45)	1 (0.98)	1 (2.86)	0.2
**Within 30-day post-TAVR outcomes**					
NYHA ≥ Class III (%)	173 (21.68)	132 (19.97)	26 (25.49)	15 (42.86)	<0.01
Myocardial infarction (%)	1 (0.13)	1 (0.15)	0 (0.00)	0 (0.00)	0.99
Stroke (%)	4 (0.50)	3 (0.45)	1 (0.98)	0 (0.00)	0.53
Disabling stroke (%)	3 (0.38)	2 (0.30)	1 (0.98)	0 (0.00)	0.43
Bleeding (%)	14 (1.75)	14 (2.12)	0 (0.00)	0 (0.00)	0.39
Life threatening bleeding (%)	2 (0.25)	2 (0.30)	0 (0.00)	0 (0.00)	0.99
New permanent pacemaker (%)	15 (1.88)	15 (2.27)	0 (0.00)	0 (0.00)	0.34
New atrial fibrillation (%)	10 (1.25)	6 (0.91)	4 (3.92)	0 (0.00)	0.05
Renal dysfunction (%)	2 (0.25)	1 (0.15)	0 (0.00)	1 (2.86)	0.1

AR, aortic regurgitation; AV, atrioventricular; N, number; NYHA, New York Heart Association; TAVR, transcatheter aortic valve replacement. Circulatory collapse was defined as a patient being in a status with mean arterial pressure ≤65 mmHg or receiving vasopressors.

### Independent predictors of NT-proBNP trajectory

The predictors of NT-proBNP trajectory class 3 are displayed in Table 3. In univariate analysis, factors including STS scoring, NYHA ≥ Class III, Prior MI, Prior PCI, baseline levels of NT-proBNP and eGFR, baseline LVEF, peak aortic velocity, mean pressure gradient, BAV, perioperative circulation collapse, and post-TAVR renal dysfunction were associated with NT-proBNP trajectory class 3 in TAVR recipients. After using multivariate logistic regression analysis, NYHA ≥ Class III (OR 2.64, 95% CI 1.07–6.51, *P* = 0.033), Prior PCI (OR 6.18, 95% CI 2.26–17.01, *P* < 0.001), baseline levels of NT-proBNP (OR 1.11 per 1,000 pg/ml increase, 95% CI 1.07–1.16, *P* < 0.001) and eGFR (OR 0.96, 95% CI 0.94–0.99, *P* = 0.008) were independently associated with NT-proBNP trajectory class 3 after TAVR.

**TABLE 3:** Multivariable predictors of NT-proBNP trajectory class three after TAVR.

Variables	Univariable		Multivariable-adjusted	
	**OR (95% CI)**	***P*-value**	**OR (95% CI)**	***P*-value**
**Baseline characteristics**				
Age (y)	1.021 (0.973–1.074)	0.4081		
Male	1.131 (0.571–2.306)	0.727		
BMI (kg/m2)	0.908 (0.818–1.003)	0.0626		
STS (%)	1.08 (1.045–1.119)	<0.0001		
NYHA ≥ Class III	2.872 (1.415–5.713)	0.0028	2.643 (1.069–6.508)	0.0332
Dyslipidemia	0.762 (0.256–1.836)	0.58		
Hypertension	0.813 (0.41–1.608)	0.5498		
Diabetes	0.486 (0.143–1.251)	0.1808		
Prior MI	8.848 (1.873–32.247)	0.0019		
Prior PCI	3.705 (1.691–7.669)	0.0006	6.179 (2.263–17.007)	0.0003
Prior stroke	1.299 (0.205–4.53)	0.7266		
PVD	1.942 (0.805–4.21)	0.111		
Atrial fibrillation	1.056 (0.389–2.43)	0.9055		
COPD	0.577 (0.194–1.387)	0.2625		
NT-proBNP (per 1,000 pg/ml increase)	1.146 (1.114–1.183)	<0.0001	1.111 (1.072–1.155)	<0.0001
eGFR (ml/min)	0.931 (0.91–0.951)	<0.0001	0.964 (0.937–0.989)	0.0084
LVEF (%)	0.944 (0.925–0.963)	<0.0001		
V max (m/s)	0.647 (0.487–0.881)	0.0035		
MPG (mm Hg)	0.975 (0.956–0.993)	0.0079		
AVA (m2)	0.591 (0.139–2.248)	0.4618		
BAV	0.347 (0.152–0.724)	0.0072		
**Procedural complications**				
New or aggravated AV block	1.568 (0.681–3.302)	0.2586		
Vascular complications	0.438 (0.024–2.101)	0.4209		
Annular rupture	NA	0.9896		
Coronary obstruction	NA	0.9881		
Circulation collapse	5.614 (1.786–14.83)	0.0011		
AR paravalvular ≥ moderate	1.504 (0.08–8.356)	0.7028		
AR transvalvular ≥ moderate	5.434 (0.274–37.975)	0.1347		
**Postoperative medications**				
Aspirin	0.632 (0.32–1.264)	0.1868		
ADP receptor inhibitor	0.608 (0.306–1.242)	0.1597		
Warfarin	0.442 (0.071–1.488)	0.2677		
Beta blocker	2.598 (0.855–6.483)	0.0594		
NOAC	NA	0.9878		
AECI/ARB	1.67 (0.694–3.608)	0.2169		
Diuretics	1.326 (0.523–2.945)	0.5151		
**Adverse events within 30-day post-TAVR**				
MI	NA	0.9896		
Stroke	NA	0.9863		
Disabling stroke	NA	0.9881		
Bleeding	NA	0.9891		
Life threatening bleeding	NA	0.9903		
New permanent pacemaker	NA	0.9887		
New atrial fibrillation	NA	0.9858		
Renal dysfunction	22.412 (0.874–574.891)	0.0291		

AECI, angiotensin converting enzyme inhibitor; ARB, angiotensin receptor blocker; AVA, aortic valve area; BAV, bicuspid aortic valve; BMI, body mass index; COPD, chronic obstructive pulmonary disease; eGFR, estimated glomerular filtration rate; LVEF, left ventricular ejection fraction; MI, myocardial infarction; MPG, mean pressure gradient; NOAC, non-vitamin K antagonist oral anticoagulants; NYHA, New York Heart Association; OR, odd ratio; PCI, percutaneous coronary intervention; PVD, peripheral vascular diseases; STS, Society of Thoracic Surgeons; V max, peak aortic velocity.

### Association between NT-proBNP trajectories and clinical outcomes

**FIGURE 2:**
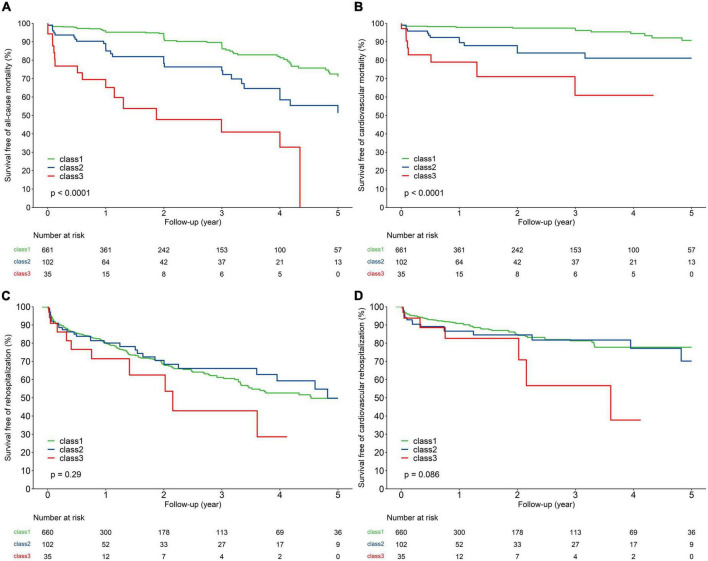
Kaplan–Meier analysis for 5-year all-cause mortality **(A)**, cardiovascular mortality **(B)**, all-cause rehospitalization **(C)**, and cardiovascular rehospitalization **(D)** of the study population according to trajectories of NT-proBNP.

Kaplan–Meier curves and crude association with clinical outcomes by NT-proBNP trajectory in the overall population are depicted in Figure 2. Compared to those with NT-proBNP trajectory class 1, patients with NT-proBNP trajectory class 2 had a more than 2.3-fold risk of 5-year all-cause death and 3.4-fold risk of cardiac death, while patients with NT-proBNP trajectory class 3 had a more than 6.6-fold risk of all-cause death and 8.8-fold risk of cardiac death. By contrast, there were no significant differences in 5-year hospitalization rates among the groups (cardiovascular and all-cause reasons).

After adjustment for baseline characteristics, peri-procedural complications, and 30-day post-TAVR outcomes, patients with NT-proBNP trajectory class 2 (HR 1.90, 95% CI 1.03–3.52, *P* = 0.04) and class 3 (HR 5.70, 95% CI 2.45–13.23, *P* < 0.01) were associated with a higher risk of 5-year all-cause mortality compared to patients with NT-proBNP trajectory class 1. Meanwhile, patients with NT-proBNP trajectory class 3 experienced an increased risk of cardiovascular mortality (HR 4.30, 95% CI 1.32–14.03, *P* = 0.02). Nevertheless, there was no significant association between NT-proBNP trajectories and all-cause and cardiovascular hospitalization (Table 4).

**TABLE 4:** Association between NT-proBNP trajectories and 5-year outcomes.

	Unadjusted		Adjusted model 1^a^		Adjusted model 2^b^		Adjusted model 3^c^	
	**HR (95 CI%)**	***P*-value**	**HR (95 CI%)**	***P*-value**	**HR (95 CI%)**	***P*-value**	**HR (95 CI%)**	***P*-value**
**All-cause mortality**								
Class 1	Ref.		Ref.		Ref.		Ref.	
Class 2	2.303 (1.464–3.622)	<0.01	1.887 (1.048–3.398)	0.03	1.924 (1.063–3.483)	0.03	1.902 (1.028–3.519)	0.04
Class 3	6.612 (3.803–11.496)	<0.01	6.263 (2.912–13.47)	<0.01	6.159 (2.792–13.585)	<0.01	5.695 (2.452–13.229)	<0.01
**CV mortality**								
Class 1	Ref.		Ref.		Ref.		Ref.	
Class 2	3.448 (1.724–6.895)	<0.01	2.481 (1.03–5.977)	0.04	2.653 (1.088–6.47)	0.03	2.295 (0.888–5.933)	0.09
Class 3	8.836 (3.898–20.028)	<0.01	7.174 (2.307–22.31)	<0.01	7.349 (2.249–24.019)	<0.01	4.304 (1.321–14.026)	0.02
**Rehospitalization**								
Class 1	Ref.		Ref.		Ref.		Ref.	
Class 2	0.93 (0.627–1.379)	0.72	0.772 (0.459–1.299)	0.33	0.756 (0.454–1.258)	0.28	0.728 (0.43–1.233)	0.24
Class 3	1.585 (0.861–2.918)	0.14	1.226 (0.551–2.731)	0.62	1.087 (0.494–2.392)	0.84	1.001 (0.438–2.289)	0.99
**CV rehospitalization**								
Class 1	Ref.		Ref.		Ref.		Ref.	
Class 2	1.241 (0.722–2.133)	0.43	1.126 (0.498–2.544)	0.78	1.076 (0.484–2.393)	0.86	0.929 (0.413–2.087)	0.86
Class 3	2.296 (1.056–4.991)	0.04	1.883 (0.613–5.782)	0.27	1.546 (0.511–4.678)	0.44	1.268 (0.397–4.054)	0.69

^a^Model 1 adjusted for baseline variables including age, sex, body mass index, Society of Thoracic Surgeons score, diabetes, hypertension, chronic obstructive pulmonary disease, estimated glomerular filtration rate, prior stroke, atrial fibrillation/flutter, left ventricular ejection fraction, New York Heart Association class and NT-proBNP levels.

^b^Model 2 adjusted for baseline variables plus procedural complications including New or aggravated atrioventricular block, vascular complications, annular rupture, coronary obstruction, circulation collapse, aortic regurgitation paravalvular ≥ moderate and aortic regurgitation transvalvular ≥ moderate.

^c^Model 3 adjusted for baseline variables plus procedural complications plus 30-day post-TAVR outcomes including New York Heart Association ≥ Class III, myocardial infarction, stroke, disabling stroke, bleeding, life threatening bleeding, new permanent pacemaker, new atrial fibrillation, renal dysfunction.

**FIGURE 3:**
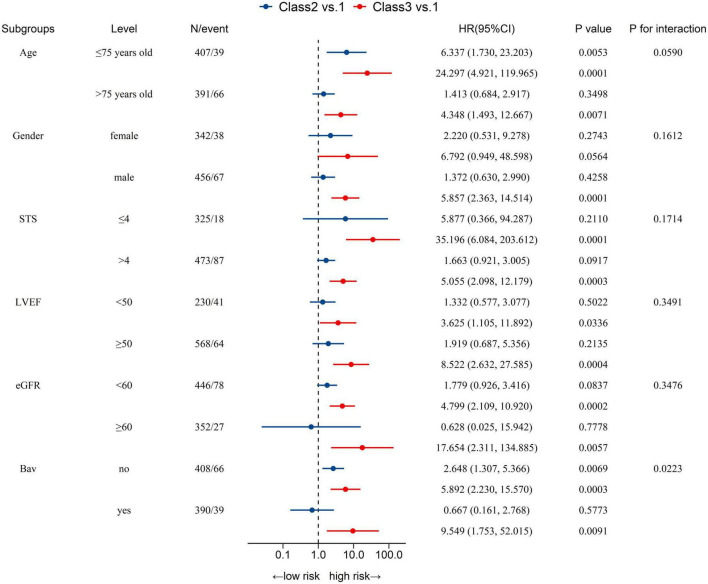
Association between trajectories of NT-proBNP and 5-year all-cause mortality after TAVR in prespecified subgroups.

As mentioned above, patients with NT-proBNP trajectory class 2 and class 3 were associated with a higher (Citation) risk of all-cause mortality than their counterparts with class 1. Subgroup analyses further demonstrated that the association between NT-proBNP trajectories and all-cause mortality was consistent regardless of age, STS scores, LVEF, eGFR, or the presence or absence of BAV (Figure 3). Nevertheless, there was an interaction by age and BAV. It suggested that the association between NT-proBNP trajectories and all-cause mortality was more significant in younger and non-BAV individuals.

## Discussion

In this study, a key finding is that we identified three distinct NT-proBNP trajectories based on their trend over time using a large cohort of severe AS patients and evaluated their association with 5-year clinical outcomes following TAVR. Patients with NT-proBNP trajectory class 2 and class 3 had an increased 5-year risk of all-cause and cardiovascular mortality. After multivariate adjustments, the risk of all-cause mortality was significantly higher in patients with NT-proBNP trajectory class 2 (HR 1.90, 95% CI 1.03–3.52, *P* = 0.04) and trajectory class 3 (HR 5.70, 95% CI 2.45–13.23, *P* < 0.01) compared to trajectory class 1. On the other hand, there were no differences in all-cause and cardiovascular hospitalization rates among the trajectories. Our findings underline the prognostic value of NT-proBNP trajectories for TAVR recipients. In addition to the baseline level of NT-proBNP, its trajectory following TAVR may have further clinical implications.

### NT-proBNP trajectories after TAVR

Studies considering the serial changes of NT-proBNP after the TAVR procedure are still limited. Previous studies have demonstrated that the dynamic profile of NT-proBNP differed between TAVR recipients (11, 16). Until now, very little was found in the literature on the different trajectories or evolution of NT-proBNP in patients undergoing TAVR. The most interesting finding in our study is that three distinct NT-proBNP trajectories were identified within 30 days following TAVR in 798 AS patients. 82.8% of patients were classified into NT-proBNP trajectory class 1, with consistently low NT-proBNP levels from baseline to post-TAVR period. As for patients with high NT-proBNP levels at baseline, the recovery of NT-proBNP levels was not as complete as expected following TAVR. Notably, 4.4% of the patients keep up high levels of NT-proBNP despite mechanical unloading of the heart by TAVR. This finding agrees with previous studies showing that BNP levels changed following TAVR according to its baseline levels (17). Mean levels remained separated between groups based on baseline levels during the 2-year follow-up. It implied that some degree of myocardial stress was retained despite the LV outflow stenosis resolution post-TAVR. Although the heterogeneity hampers direct comparison in study populations and cutoff values, our data suggest that serial NT-proBNP monitoring before and after TAVR may be best practice for an individual undergoing TAVR. It contributes to risk estimation more accurately than from a single measure of baseline levels. Such a strategy can also address other conditions that affect natriuretic peptide levels and prognosis, including ischemic heart disease, atrial fibrillation, and kidney dysfunction.

### Prognostic value of the NT-proBNP trajectories

Prior studies examining the relationship between natriuretic peptide levels and post-TAVR clinical outcomes have been inconsistent and yielded conflicting results (18–21). Natriuretic peptide levels fluctuated with age, sex, and kidney function, and cutoffs were inconsistent with inadequate performance (22, 23). NT-proBNP is proposed as a better marker for prognostication with a longer half-life and better stability than BNP (24). While much attention has been paid to the baseline NT-proBNP as a predictor of outcomes before an intervention, less emphasis has been placed on the post-TAVR NT-proBNP levels or their trajectories (10, 25). To our knowledge, we are the first to evaluate NT-proBNP trajectories in the AS population undergoing TAVR, and provided a biomarker lens through which to see their residual risk after TAVR.

Our findings are consistent with previous studies on residual NT-proBNP elevation after TAVR, in that postoperative NT-proBNP level was associated with more clinical events and worse quality of life (19, 26). Further, our data suggest that the contextual assessment of the NT-proBNP profiles in the form of longitudinal trajectories could provide a more nuanced identification of patients at high risk. We demonstrate that NT-proBNP levels evolve differently from baseline to postoperative period with distinct trajectories and independently associated with subsequent clinical outcomes. We noted that individuals with NT-proBNP trajectories class 2 and class 3 were at a significantly higher risk of post-TAVR mortality. These findings highlight that many patients with ongoing elevations in NT-proBNP levels are subject to untoward consequences even after unloading the heart with TAVR.

### Underlying mechanisms of the NT-proBNP trajectories

Several potential mechanisms, or their interplay might account for the observed association between NT-proBNP trajectories and post-TAVR outcomes. While the mechanisms are similar to that of underlying heart failure, there is undoubtedly some distinctive pathophysiology stemming from the chronic pressure overload that is suddenly relieved by TAVR. Natriuretic peptide levels correlate with the deleterious pathophysiology in AS, including maladaptive hypertrophic remodeling, impaired systolic and diastolic function, increased wall stress, and volume overload (27). Even after TAVR, they often do not completely reverse, yielding substantial residual risk related to ongoing heart failure (28). Additionally, an elevated NT-proBNP level persisting after TAVR is likely the result of comprehensive factors, including residual cardiac hypertrophy/fibrosis, impaired cardiac function, volume overload, and other concomitant diseases (29, 30).

If the intervention is postponed until NT-proBNP levels rise substantially, some irreversible cardiac damage may have already occurred (31). Prior studies speculated that high levels of BNP reflect the transition from physiological to maladaptive hypertrophy, serving as a marker for the irreversibility of the myocardial changes despite valve replacement (32–34). As mentioned above, a continued rise in NT-proBNP levels after TAVR was associated with a worse prognosis. Thus, it is plausible that efforts to target the etiology driving elevation of NT-proBNP may influence clinical outcomes in TAVR recipients. Accordingly, earlier valve replacement (before pathological hypertrophic remodeling becomes irreversible) and optimal medical therapy could be reasonable for lower post-TAVR NT-proBNP levels and better clinical outcomes. This hypothesis can be tested in ongoing trials exploring the effective concomitant therapy and optimal timing of TAVR in AS patients.

### Clinical implications

Regardless of the underlying etiology, NT-proBNP trajectories may theoretically identify TAVR recipients with a better prognosis. Our findings implied different evolution of NT-proBNP levels in TAVR recipients and the necessity of reevaluating NT-proBNP following TAVR. This finding has important implications: it is necessary to reassess residual NT-proBNP within short periods after TAVR. It can be leveraged to gain long-term benefits for better risk stratification and prognostication in AS patients undergoing TAVR. Nevertheless, as our cohort included only severe AS patients, these results cannot be generalized to all AS patients. The implications of these findings are undoubtedly plausible but need to be confirmed. Further investigation is warranted to understand the underlying mechanisms and determine whether steps taken to mitigate the rise in NT-proBNP levels may improve the clinical outcomes of TAVR recipients, particularly as TAVR continues to expand into younger, lower-risk populations (with fewer non-cardiac comorbidities).

## Limitations

First, we acknowledge that our data comes from a relatively small, single-center, observational study. Given the different number of patients in three groups, our results should be interpreted with caution. Although our findings regarding the association of mortality and NT-proBNP trajectories remain statistically significant after multivariable adjustment, we cannot rule out the possibility that other unmeasured factors confound the analysis. To minimize heterogeneity in our study cohort, we did not include those treated with surgical aortic valve replacement or valve-in-valve TAVR, limiting the generalizability to those patients. It is worth mentioning that our preliminary findings suggest procedural complications and adverse events within 30-day after TAVR have little effect on NT-proBNP trajectory in TAVR recipients. This point should be interpreted with caution in case of potential underestimation. The reason of statistical insignificance might lie partly in the relatively low event rate of adverse events in our cohort and the exclusion of patients who died within 30 days after TAVR. Furthermore, patients without an NT-proBNP measurement were excluded from our analysis at any time. Undeniably, patients excluded may have better clinical outcomes with less cause to measure NT-proBNP. Lastly, our studies were fitted based on assigned trajectories. They did not take into account the uncertainty in the class membership of each individual, which means that the variance estimates from our models might be underestimated; however, given that the posterior probabilities of class membership were universally high and the robustness of our findings for the trajectories across several analyses, it is unlikely that this would affect the general conclusions.

## Conclusion

Taken together, different trajectories of NT-proBNP identified by repeated measurement post-TAVR were associated with 5-year clinical outcomes in TAVR recipients, with NT-proBNP trajectory class 2 and class 3 experiencing an increased 5-year risk of all-cause and cardiovascular mortality. Our findings implied different evolution of NT-proBNP levels in TAVR recipients and the utility of reevaluating NT-proBNP following TAVR. Future studies are warranted to unravel the biological underpinnings of these associations and the potential for using NT-proBNP trajectories (as opposed to single assessment) as a strategy to identify TAVR recipients at high risk of poor prognosis. Whether efforts to mitigate post-TAVR NT-proBNP levels through earlier timing of TAVR or intensifying medical therapy yields better clinical outcomes requires further investigation.

## Data availability statement

The original contributions presented in this study are included in this article/Supplementary material, further inquiries can be directed to the corresponding authors.

## Ethics statement

The studies involving human participants were reviewed and approved by the Medical Ethics Committee of the Second Affiliated Hospital of Zhejiang University. The patients/participants provided their written informed consent to participate in this study.

## Author contributions

ZP and JW contributed to the conception of the study. PH and HL contributed to analysis and manuscript preparation. YZ and QZ performed the data analyses and wrote the manuscript. XPL and XBL helped perform the analysis with constructive discussions. All authors contributed to the article and approved the submitted version.

## Supplementary material

Supplementary Figure 1 Flowchart of patient enrollment, including study inclusion and exclusion criteria.

Supplementary Figure 2 Kaplan–Meier analysis for 5-year all-cause mortality **(A)**, cardiovascular mortality **(B)**, all-cause rehospitalization **(C)**, and cardiovascular rehospitalization **(D)** of the study population according to NT-proBNP levels before hospital discharge.
