# Supplementary figures and images for: NT-proBNP trajectory after transcatheter aortic valve replacement and its association with 5-year clinical outcomes
Source: Front Cardiovasc Med. 2023 Feb 17;10:1098764. doi: 10.3389/fcvm.2023.1098764 (PMC9981663; doi:10.3389/fcvm.2023.1098764)

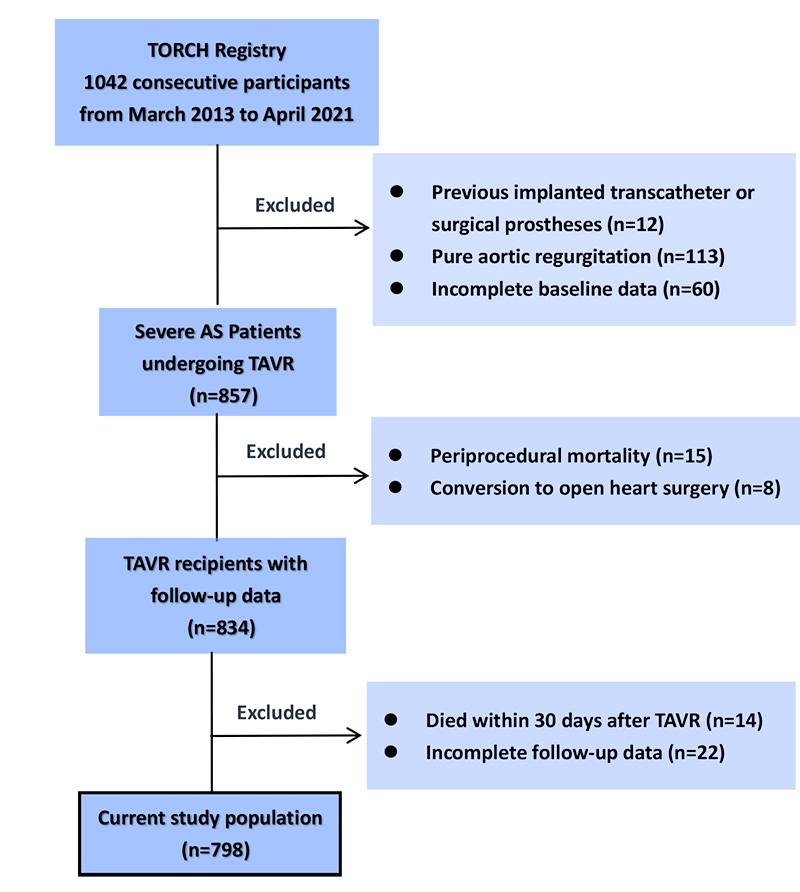

Supplement: Supplementary Figure 1 — Flowchart of patient enrollment, including study inclusion and exclusion criteria. [file Image_1.TIF]
