# Supplementary material for: NT-proBNP trajectory after transcatheter aortic valve replacement and its association with 5-year clinical outcomes
Source: Front Cardiovasc Med. 2023 Feb 17;10:1098764. doi: 10.3389/fcvm.2023.1098764 (PMC9981663; doi:10.3389/fcvm.2023.1098764)
Supplement: Supplementary file 3 [file Table_1.DOCX]

**Supplemental Table 1. Univariable and multivariable Cox proportional hazards analysis on 5-year survival for TAVR recipients**

|  | Univariable | | Multivariable‐adjusted | |
| --- | --- | --- | --- | --- |
| Variables | **HR (95% CI)** | **P-value** | **HR (95% CI)** | **P-value** |
| Age (y) | 1.060 (1.027 - 1.094) | <0.001 |  |  |
| Male | 1.210 (0.813 - 1.802) | 0.348 |  |  |
| Smoker | 1.082 (0.643 - 1.821) | 0.767 |  |  |
| BMI (kg/m2) | 0.926 (0.874 - 0.981) | 0.009 |  |  |
| STS (%) | 1.076 (1.061 - 1.092) | <0.001 | 1.061 (1.041 - 1.082) | <0.001 |
| NYHA ≥ Class III | 0.994 (0.662 - 1.491) | 0.976 |  |  |
| Dyslipidemia | 0.825 (0.513 - 1.328) | 0.429 |  |  |
| Hypertension | 1.067 (0.725 - 1.571) | 0.741 |  |  |
| Diabetes | 1.153 (0.726 - 1.832) | 0.546 |  |  |
| Prior MI | 0.433 (0.060 - 3.108) | 0.406 |  |  |
| Prior PCI | 1.239 (0.705 - 2.175) | 0.456 |  |  |
| Prior stroke | 2.138 (1.114 - 4.104) | 0.022 | 1.757 (0.891 - 3.464) | 0.104 |
| PVD | 1.827 (1.202 - 2.777) | 0.005 | 1.993 (1.275 - 3.116) | 0.002 |
| Atrial fibrillation | 2.426 (1.606 - 3.664) | <0.001 |  |  |
| COPD | 1.243 (0.802 - 1.927) | 0.330 |  |  |
| NT-proBNP  (per 1000 pg/ml increase) | 1.000 (1.000 - 1.000) | <0.001 |  |  |
| eGFR (ml/min) | 0.976 (0.967 - 0.985) | <0.001 |  |  |
| LVEF (%) | 0.982 (0.970 - 0.995) | 0.007 | 1.020 (1.005 - 1.036) | 0.011 |
| V max (m/sec) | 0.696 (0.570 - 0.849) | <0.001 |  |  |
| MPG (mm Hg) | 0.982 (0.971 - 0.994) | 0.002 |  |  |
| AVA (m2) | 0.723 (0.306 - 1.707) | 0.460 |  |  |
| BAV | 0.654 (0.440 - 0.972) | 0.036 |  |  |
| New or aggravated AV block | 0.961 (0.456 - 2.022) | 0.916 |  |  |
| Vascular complications | 2.294 (1.193 - 4.411) | 0.013 |  |  |
| Circulation collapse | 5.779 (2.881 - 11.591) | <0.001 | 4.471 (2.087 - 9.577) | <0.001 |
| MI | 3.647 (0.506 - 26.266) | 0.199 |  |  |
| Stroke | 0.831 (0.116 - 5.975) | 0.854 |  |  |
| Bleeding | 2.657 (0.837 - 8.434) | 0.097 |  |  |
| New permanent pacemaker | 1.561 (0.384 - 6.341) | 0.533 |  |  |
| New atrial fibrillation | 0.566 (0.079 - 4.059) | 0.571 | 1.495 (0.959 - 2.332) | 0.076 |
| Renal dysfunction | 4.749 (0.659 - 34.209) | 0.122 |  |  |
| NT-proBNP trajectories class 1 | reference |  | reference |  |
| NT-proBNP trajectories class 2 | 2.303 (1.464 - 3.622) | <0.001 | 1.669 (0.975 - 2.856) | 0.062 |
| NT-proBNP trajectories class 3 | 6.612 (3.803 - 11.496) | <0.001 | 4.391 (2.355 - 8.189) | <0.001 |
